# Identification of Characteristic Fatty Acids to Quantify Triacylglycerols in Microalgae

**DOI:** 10.3389/fpls.2016.00162

**Published:** 2016-02-22

**Authors:** Pei-Li Shen, Hai-Tao Wang, Yan-Fei Pan, Ying-Ying Meng, Pei-Chun Wu, Song Xue

**Affiliations:** ^1^Marine Bioengineering Group, Department of Biotechnology, Dalian Institute of Chemical Physics, Chinese Academy of SciencesDalian, China; ^2^University of Chinese Academy of SciencesBeijing, China; ^3^Department of Environmental Science and Engineering, College of Environment and Chemical Engineering, Dalian UniversityDalian, China; ^4^School of Life Science and Biotechnology, Dalian University of TechnologyDalian, China

**Keywords:** microalgae, triacylglycerols, neutral lipids, characteristic fattyacids, biofuels

## Abstract

The fatty acid profiles of lipids from microalgae are unique. Polyunsaturated fatty acids are generally enriched in polar lipids, whereas saturated and monounsaturated fatty acids constitute the majority of fatty acids in triacylglycerols (TAG). Each species has characteristic fatty acids, and their content is positively or negatively correlated with TAGs. The marine oleaginous diatom *Phaeodactylum tricornutum* was used as the paradigm to determine the quantitative relationship between TAG and characteristic fatty acid content. Fatty acid profiles and TAG content of *Phaeodactylum tricornutum* were determined in a time course. C16:0/C16:1 and eicosapentaenoic acid (EPA, C20:5n3) were identified as characteristic fatty acids in TAGs and polar lipids, respectively. The percentage of those characteristic fatty acids in total fatty acids had a significant linear relationship with TAG content, and thus, the correlation coefficient presenting *r*^2^ were 0.96, 0.94, and 0.97, respectively. The fatty acid-based method for TAG quantification could also be applied to other microalgae such as *Nannochloropsis oceanica* in which the *r*^2^ of C16:0 and EPA were 0.94 and 0.97, respectively, and in *Chlorella pyrenoidosa r*^2^-values for C18:1 and C18:3 with TAG content were 0.91 and 0.99, repectively. This characteristic fatty acid-based method provided a distinct way to quantify TAGs in microalgae, by which TAGs could be measured precisely by immediate transesterification from wet biomass rather than using conventional methods. This procedure simplified the operation and required smaller samples than conventional methods.

## Introduction

Microalgae are potential triacylglycerol (TAG) resource for biofuel production (Chisti, 2007; Williams and Laurens, 2010). Despite analyses indicating that biofuels may be able to substitute for petroleum-derived transport fuels in the future (Chisti, 2008; Sander and Murthy, 2010), biofuels from microalgae have not yet been commercialized due to high cost (Williams and Laurens, 2010). Increasing lipid production, especially TAGs, is critical to reduce the cost of microalgae-based biofuels. Therefore, a suitable method to quantify the lipid content in microalgae is necessary.

Oil content in microalgae is commonly assessed using a gravimetric method. Oil in microalgae is extracted with organic solvents, using the general rule of “like dissolves like” (Bligh and Dyer, 1959; Ryckebosch et al., 2012; Axelsson and Gentili, 2014). However, many cellular components, such as pigments and unknown molecules, may be co-extracted with lipids due to their similar polarity, and these components may fluctuate during cultivation. Therefore, this method is unsuitable for assessing lipid production (Wang et al., 2009). Furthermore, this method requires a relatively large amount of sample, usually approximately 100 mg, and prevents conducting a time-course metabolic study of microalgae. Thin-layer chromatography (TLC) followed by capillary gas chromatography-flame ionization detection (GC-FID) is another method used to quantify lipids. This method separates lipids into individual classes by TLC after extraction from the cell using organic solvents. The lipids are then converted into fatty acid methyl esters (FAMEs) by GC-FID quantification. Due to the sensitivity of GC-FID, a few milligrams are sufficient for quantification. However, the method is time and labor intensive (Chen et al., 2009). High-performance liquid chromatography-mass spectrometry (HPLC-MS) has been used to detect lipids directly (MacDougall et al., 2011; Kind et al., 2012), but this approach is not routinely done due to the complex sample compositions. In addition, mutant screening is a powerful method to acquire an in-depth understanding of lipid metabolism (Doan and Obbard, 2012; Li et al., 2012; Manandhar-Shrestha and Hildebrand, 2013). Development of convenient and high-throughput methods to quantify lipids in microalgae has garnered increasing attention due to the labor-intensive nature of mutant screening (Terashima et al., 2015).

Lipids can be categorized into neutral and polar lipids, and neutral lipids are primarily referred to as TAGs. The fatty acid profiles in both microalgae and plants are unique to specific lipid classes. Neutral lipids mainly contain saturated (SFAs) and monounsaturated fatty acids (MUFAs), such as C16:0, C16:1, and C18:1, and more than 50% of C18:1 and C16:0 fatty acids that accumulate in the model algae *Chlamydomonas reinhardtii* are in the TAGs (Siaut et al., 2011). Contrarily, the neutral lipids in *Chlamydomonas reinhardtii*, polyunsaturated fatty acids (PUFAs), are enriched in polar lipids, such as C16:4, C18:3, and eicosapentaenoic acid (EPA, C20:5n3). C16:4 and C18:3 comprise the bulk of fatty acids in monogalactosyldiacylglycerols (MGDG), which account for more than 80% C16:4 and C18:3 of the total fatty acid content of *Chlamydomonas reinhardtii* (Zäuner et al., 2012). In the model plant *Arabidopsis thaliana*, C16:3 and C18:3 comprise the majority of MGDG and the main fatty acids in TAGs are C16:0, C18:0, and C18:1 (Fan et al., 2013). The total fatty acid profiles depend on the distinct fatty acid composition of the lipids. A strong correlation (*r*^2^ = 0.986) has been reported between the 16:0/16:4 ratio in FAMEs derived directly from algal cells and the TAG/total acyl group ratio in *Chlamydomonas reinhardtii* (Liu et al., 2013). More recently, we have reported that the neutral lipid content in *Isochrysis zhangjiangensis* can be quantified based on the C18:1 or C18:4 content (Wang et al., 2014). Therefore, we propose that TAG content can be quantified using specific fatty acids.

Here, the marine microalgae *Phaeodactylum tricornutum*, which was an ideal candidate for producing biodiesel, was studied as an example to support this hypothesis. First, the principles of characteristic fatty acid identification were discussed based on an analysis of the fatty acid profile of individual lipid in *Phaeodactylum tricornutum*. Subsequently, the relationship between the characteristic fatty acids and the TAG content was analyzed. This procedure was also applied to different microalgal species, such as *Nannochloropsis oceanica* and *Chlorella pyrenoidosa*, to expand its application.

## Materials and methods

### Strains and culture conditions

The marine microalgae *Phaeodactylum tricornutum* was provided by Dr. Weidong Liu of the Liaoning Institute of Marine Fisheries. The marine microalgae *Nannochloropsis oceanica* IMET1 was provided by Dr. Jian Xu of the Qingdao Institute of Bioenergy and Bioprocess Technology, Chinese Academy of Sciences. *Chlorella pyrenoidosa* was obtained from the Freshwater Algae Culture Collection of the Institute of Hydrobiology, Chinese Academy of Sciences.

To identify the characteristic fatty acids, enriched f/2 medium, which contained three-fold the concentration of all ingredients, was inoculated with *Phaeodactylum tricornutum* as described by Feng et al. (2011). The medium was supplemented with sodium nitrate (N+), and the algae were stressed by omitting sodium nitrate (N−).

To correlate the characteristic fatty acids with neutral lipids, *Phaeodactylum tricornutum* and *Nannochloropsis oceanica* IMET1 were cultured under N− (Meng et al., 2015). *Chlorella pyrenoidosa* was cultured in BG11 medium without sodium nitrate to generate the N− condition.

Total fatty acid profiles and TAG content of three species were determined in a time course. The sampling time of the three species were 0, 6, and 18 h of *Phaeodactylum tricornutum*; 0, 2, 4, 7, 9, 12, and 14 h of *Nannochloropsis oceanica* IMET1; and 0, 1, 5, 6, and 8 days of *Chlorella pyrenoidosa*, respectively. Three replicates were taken for each time point. Each data point was a biological replicate (*n* = 3).

An average irradiance of 70 μmol photons m^−2^ s^−1^ was provided by continuous illumination with cool white fluorescent lamps. Temperature was maintained at 25 ± 2°C.

### Lipids extraction and fatty acids analysis

Cells were collected by centrifugation, and lipids were extracted according to the method described by Wang and Benning (2011) with slight modifications. A 300-μL aliquot of extraction solvent composed of methanol, chloroform, and formic acid (20:10:1, v/v/v) was added to approximately 5 mg biomass (dry weight). After shaking vigorously for 5 min, 150 μL of 0.2 M phosphoric acid and 1 M potassium chloride were added and vortexed for 1 min. Phase separation was achieved after centrifugation at 12,000 × g at room temperature for 5 min; the lipids dissolved in the lower chloroform phase were spotted onto TLC silica plates. Glycolipids (GL) and phospholipids (PL) were separated using a solvent system of acetone:toluene:water int the ration 91:30:7.5 mL, respectively. TAGs were separated with hexane:diethyl ether:acetic acid in the ratio 85:15:1, v/v/v, respectively.

The lipids separated by TLC or a microalgal biomass was added to methanol (2% H_2_SO_4_) and incubated for 1 h at 70°C to prepare the FAMEs. After methylation, deionized water and hexane were added to extract the FAMEs. C17:0-TAG was added as an internal standard for quantification. The FAMEs were identified qualitatively by mass spectroscopy and quantitatively by gas chromatography with a FID. The FAMEs of samples were detected on a 7890B gas chromatograph with a DB-23 capillary column (30 m × 0.32 mm × 0.25 μm; Agilent Technologies, Santa Clara, CA, USA) and a FID. The injector temperature was 260°C with a split ratio of 50:1. The initial column temperature was 130°C and was maintained for 1 min. The temperature was then increased to 170°C at a rate of 10°C min^−1^, followed by an increase to 215°C at a rate of 2.8°C min^−1^. The temperature was then maintained at 215°C for 1 min.

### Statistical data analysis

Correlation coefficients between characteristic fatty acids and TAG content were calculated using Origin Pro software, version 8.0 (Origin Lab, Hampton, Massachusetts, USA). Statistical significance in each result was calculated using the Two-tailed by SPSS 18.0 for windows (IBM, Armonk, New York City, USA), where the significance *P* < 0.01.

## Results

### Identification of characteristic fatty acids in *phaeodactylum tricornutum*

Characteristic fatty acids were present in different classes of lipids, and it was necessary to identify the characteristic fatty acids to accurately quantify TAGs. The fatty acid profiles of different lipid classes and TAG content were determined under both N+ and N− to identify the characteristic fatty acids. The total lipids (TLs) extracted from *Phaeodactylum tricornutum* cells were separated into GLs, PLs, and TAGs by TLC. All lipids except for TAGs were polar lipids. The fatty acid profile of individual lipid and TLs that were extracted from cells cultured under N+ and N− were analyzed (Table 1). PUFAs were the main fatty acids in GLs and PLs both under N+ and N−. EPA was the most abundant fatty acid in GLs and PLs and accounted for approximately 25% of the total fatty acids from these lipids. Therefore, EPA was the characteristic fatty acid of the polar lipids. TAGs were enriched with SFAs and MUFAs rather than PUFAs, such as C16:0, which was identified as a characteristic fatty acid. C16:1 almost evenly distributed across the aforementioned lipid classes and constituted 32–36% of the fatty acids in all lipid classes under N+. TAGs were specifically enriched with C16:1 under the N−, which was the most abundant fatty acid, accounting for almost 50% of fatty acid content. Although C16:1 content in PLs decreased dramatically, it remained largely unchanged in GLs and accounted for about 30% of its total fatty acids. Therefore, C16:1 was not specific to TAGs and could not serve as a characteristic fatty acid of TAGs.

**Table 1:** **Fatty acid profiles of different lipid components (means ± *SD, n* = 3) in *Phaeodactylum tricornutum* under nitrogen replete (N+) and nitrogen deplete (N−) were obtained by capillary FID-GC**.

**Lipids**	**TL**		**GL**		**PL**		**TAG**	
**Medium**	**N+**	**N−**	**N+**	**N−**	**N+**	**N−**	**N+**	**N−**
C14:0	3.0±0.3	4.2±0.1	3.1±0.7	5.4±0.6	2.9±0.5	3.2±0.3	2.8±0.2	4.1±0.0
C16:0	11.3±0.4	27.5±0.6	9.0±0.2	17.8±0.3	11.9±1.0	15.7±0.3	23.3±1.9	32.4±0.1
C16:1n7	35.5±1.3	41.9±1.0	35.0±1.3	32.5±0.3	37.1±1.1	16.9±0.7	33.8±2.6	48.9±0.3
C16:2n4	5.8±0.1	1.9±0.1	7.2±0.2	4.2±0.0	0.8±0.1	0.2±0.1	12.3±0.6	1.5±0.1
C16:3n4	8.4±0.2	2.7±0.1	13.6±0.3	10.6±0.5	0.7±0.2	0.4±0.1	0.3±0.4	0.6±0.0
C18:0	2.5±0.5	1.6±0.4	0.8±0.3	1.7±0.8	1.3±0.6	2.1±0.5	15.4±2.5	1.5±0.2
C18:1n9	2.1±0.2	4.5±0.1	0.5±0.1	1.0±0.2	5.6±0.7	17.0±0.8	1.7±0.4	3.5±0.1
C18:1n7	0.9±0.3	1.1±0.0	0.5±0.1	0.8±0.1	2.0±0.5	1.8±0.1	0.5±0.0	1.1±0.0
C18:2n6	1.6±0.0	0.7±0.3	0.5±0.0	0.3±0.2	3.8±0.1	2.9±0.2	1.0±0.1	0.4±0.3
C18:3n6	0.3±0.0	0.4±0.0	0.3±0.0	0.0±0.1	0.5±0.0	1.0±0.1	0±0	0.4±0.0
C18:3n3	0.3±0.0	0.0±0	0.2±0.0	0±0	0.7±0.1	0±0	0.1±0.2	0.1±0
C18:4n3	0.4±0.1	0.2±0.0	0.4±0.0	0.4±0.0	0.4±0.1	0.1±0.1	0.2±0.2	0.2±0.0
C18:5n3	1.2±0.2	0.7±0.2	1.1±0.2	1.9±0.7	0.9±0.2	1.1±0.4	2.6±0.2	0.3±0.1
C20:5n3	24.3±0.6	11.8±0.8	26.9±1.2	23.4±0.4	25.5±0.9	33.1±0.3	5.2±2.2	4.7±0.1
C22:6	2.4±0.4	0.8±0.1	0.9±0.2	0.1±0.1	5.9±0.8	4.7±0.1	0.8±0.6	0.4±0.0

*TL, total lipid; GL, glycolipid; PL, phospholipid; TAG, triacylglycerol*.

The fatty acid distributions were determined in different lipid classes to evaluate the contributions of each FAME to the total fatty acid profiles (Table 2). EPA was present in polar lipids under the N+, and more than 70% of the EPA was detected in polar lipids under the N−, despite the decreased amount of EPA compared with that detected under the N+. Only about 20% of the C16:0 was detected in TAGs under the N+, but this proportion increased to 80% under the N−. Because most of the EPA was found in polar lipids, the variation in polar lipids could be represented by the proportion of characteristic fatty acids of total fatty acids. Given the inverse relationship between polar lipids and TAGs, EPA was negatively correlated with TAG content. Similar to the characteristic fatty acids of polar lipids, the proportion of TAG-characteristic fatty acids also represented changes in TAG content.

**Table 2:** **Fatty acids distribution in different lipid classes (means ± *SD, n* = 3) in *Phaeodactylum tricornutum* under nitrogen replete (N+) and nitrogen deplete (N−)**.

**Lipids**	**GL**		**PL**		**TAG**	
**Medium**	**N+**	**N−**	**N+**	**N−**	**N+**	**N−**
C14:0	61.0±9.9	26.9±2.6	29.8±8.5	8.4±2.0	9.1±1.4	64.8±3.2
C16:0	48.1±2.7	13.9±1.4	31.5±3.4	6.2±1.0	20.3±1.6	79.9±2.4
C16:1n7	59.3±2.4	16.6±1.7	31.2±1.8	4.4±0.9	9.4±0.6	79.0±2.5
C16:2n4	74.7±1.5	46.9±1.7	4.3±0.3	1.2±0.9	21±1.2	51.9±2.6
C16:3n4	97.1±0.6	84.1±0.7	2.6±0.8	1.7±0.4	0.3±0.4	14.2±1.1
C18:0	21.2±7.7	21.5±6.3	14.7±6.7	13.7±1.7	64.1±12.8	64.8±7.9
C18:1n9	13.4±1.0	4.9±0.8	78.7±0.8	41.2±3.7	8.0±0.9	53.9±4.0
C18:1n7	31.6±2.0	15.4±2.7	62.7±2.5	17.8±2.8	5.7±0.5	66.8±5.4
C18:2n6	20±1.9	7.5±5.3	73.4±1.1	57.4±23.0	6.6±0.7	35.2±17.7
C18:3n6	53.2±0.9	1.8±2.6	46.8±0.9	27.3±3.7	0±0	70.9±3.6
C18:3n3	34.0±2.25	0±0	63.1±3.2	0±0	2.8±4.0	100.0±0
C18:4n3	63.8±7.0	36.2±0.2	32.5±2.4	4.6±6.5	3.6±5.2	59.2±6.3
C18:5n3	55.2±1.2	54.5±1.9	22.7±2.4	18.0±7.9	22.2±3.4	27.5±6.7
C20:5n3	66.5±1.2	42.3±0.3	31.3±0.3	30.4±2.6	2.2±1.2	27.3±2.9
C22:6	22.3±2.4	2.7±3.8	74.3±0.2	64.0±5.0	3.3±2.4	33.3±4.2

*TL, total lipid; GL, glycolipid; PL, phospholipid; TAG, triacylglycerol*.

### Correlations between tag content and characteristic fatty acids in *phaeodactylum tricornutum*

**Figure 1:**
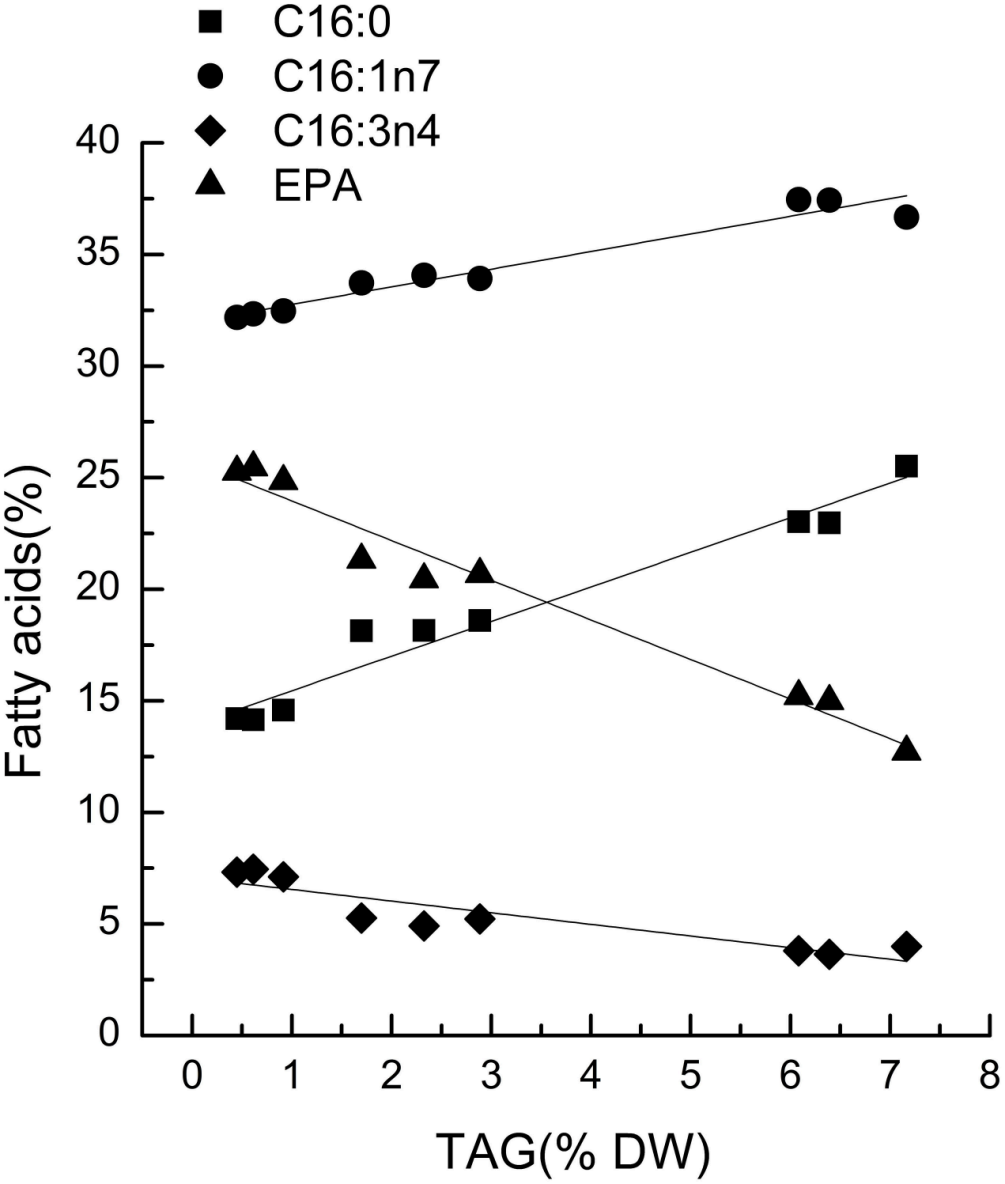
**Correlations between characteristic fatty acids and triacylglycerol (TAG) content in *Phaeodactylum tricornutum***. The *r*^2^-values for C16:0, 16:1n7, C16:3n4, and eicosapentaenoic acid (EPA, C20:5n3) were 0.96, 0.94, 0.80, and 0.97, respectively. The sampling time of *Phaeodactylum tricornutum* was 0, 6, and 18 h. Each time point was made with three replicates, which represent biological replicates (*n* = 3). The points were drawn from individual result rather than the average of the three replicates. Statistical significance in each result was calculated using the Two-tailed, *P* < 0.01.

Characteristic fatty acids had positive or negative correlations with TAGs in microalgae. The fatty acid profiles of TLs and TAG content were determined over time after nitrogen depletion in *Phaeodactylum tricornutum* to quantify the relationship between the characteristic fatty acids and TAG content. A correlation analysis revealed that fatty acid levels were highly and significantly correlated with TAG content (Figure 1). Specifically, the correlation coefficients of C16:0, C16:1, and EPA with the TAG content were 0.96, 0.94, and 0.97, respectively. The linear relationship between the characteristic fatty acids and the TAG content revealed that characteristic fatty acids could be used to quantify TAG content.

## Discussion

### Principles to identify characteristic fatty acids

As some fatty acids were correlated with TAGs in microalgae, identifying the characteristic fatty acids was the first step to quantify TAG content. In order to quantify the characteristic fatty acid accurately, there was a threshold in the amount of characteristic fatty acids. Therefore, the proportion of fatty acids of TLs was a key rule for identifying characteristic fatty acids. Almost all C16:3 were found in GLs under the N− and N+ (Table 2). However, low total fatty acid content (Table 1) made it unsuitable to quantify TAGs. The correlation coefficient between the C16:3 content in total fatty acids and TAG content was only 0.798. We set 10% as the minimum content criterion for identifying characteristic fatty acids, therey considering a 10% variation in the percentage of each fatty acid determined by immediate transesterification of wet biomass (Liu et al., 2015), and C16:3 accounted for less than10% of total fatty acids in *Phaeodactylum tricornutum*. A significant change of cellular TAG accumulation was another key point when to determine the characteristic fatty acids, and the minimum fold-change was set to 1.5. Although C16:1 was increased in the TAGs under the N−, the ~1.2-fold change in C16:1 of the total fatty acids was insufficient for quantification. Taken together, 10% total fatty acid content and a 1.5-fold change were the thresholds for defining the characteristic fatty acids.

### Expanding the characteristic fatty acid-based method to other microalgae

**Figure 2:**
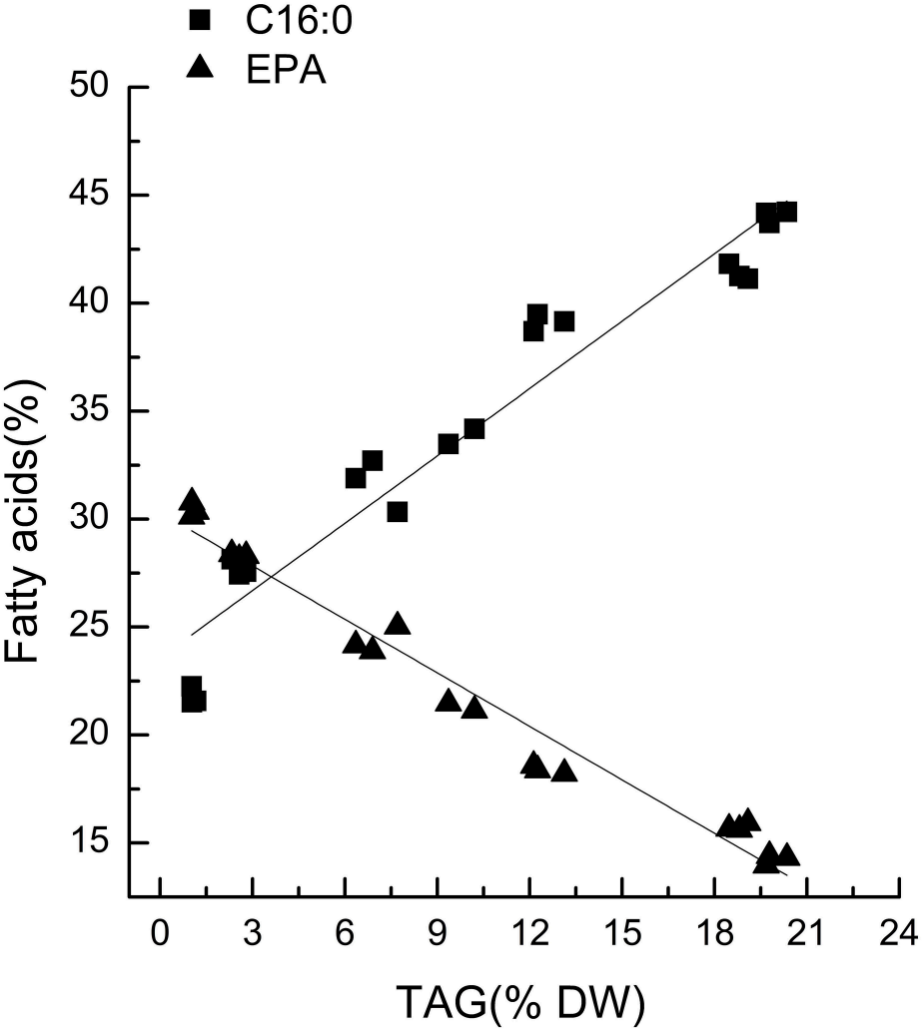
**Correlations between characteristic fatty acids and triacylglycerol (TAG) content in *Nannochloropsis oceanica* IMET1**. The *r*^2^-values for C16:0 and eicosapentaenoic acid (EPA, C20:5n3) were 0.94 and 0.97, respectively. The sampling time of *Nannochloropsis oceanica* IMET1 was 0, 2, 4, 7, 9, 12, and 14 h. Each time point was made with three replicates, which represent biological replicates (*n* = 3). The points were drawn from individual result rather than the average of the three replicates. Statistical significance in each result was calculated using the Two-tailed, *P* < 0.01.

**Figure 3:**
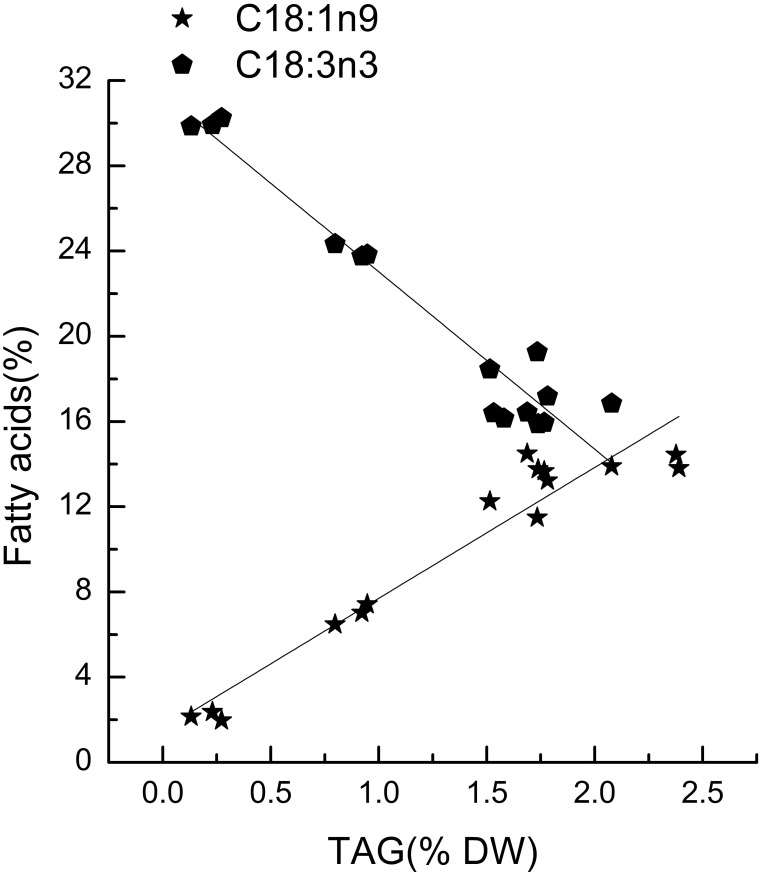
**Correlations between characteristic fatty acids and triacylglycerol (TAG) content in *Chlorella pyrenoidosa***. The *r*^2^-values for C18:1n9 and C18:3n3 were 0.91 and 0.99, respectively. The sampling time of *Chlorella pyrenoidosa* was 0, 1, 5, 6, and 8 days. Each time point was made with three replicates, which represent biological replicates (*n* = 3). The points were drawn from individual result rather than the average of the three replicates. Statistical significance in each result was calculated using the Two-tailed, *P* < 0.01.

*Chlorella pyrenoidosa* (Chlorophyta) and *Nannochloropsis oceanica* (Eustigmatophyceae) were chosen as examples to expand the characteristic fatty acid-based method to other taxa. The fatty acid profiles of total lipids and TAG content were determined in a time course after nitrogen depletion in *Nannochloropsis oceanica* (Table 3) and *Chlorella pyrenoidosa* (Table 4), as described for *Phaeodactylum tricornutum*. The principles discussed above were used to identify the characteristic fatty acids in the two species. C16:0 and EPA were determined to be characteristic fatty acids in *Nannochloropsis oceanica*. The linear relationship between the characteristic fatty acids and TAG content was shown in Figure 2. The correlation coefficients of C16:0 and EPA with TAG content were 0.94 and 0.97 in *Nannochloropsis oceanica*, respectively. Similar to *Nannochloropsis oceanica*, C18:1 and C18:3 were determined to be characteristic fatty acids in *Chlorella pyrenoidosa*. The correlation coefficients of C18:1 and C18:3 with TAG content were 0.91 and 0.99 in *Chlorella pyrenoidosa*, respectively (Figure 3). The high correlation coefficients between the characteristic fatty acids and TAG content demonstrated that the characteristic fatty acid-based method could accurately measure TAG content. In addition, two types of characteristic fatty acids, such as EPA and C18:1, were detected that were negatively or positively correlated with TAGs, respectively, because they were from GLs or TAGs, which were different sources.

**Table 3:** **Fatty acid profiles of total lipids (means ± *SD, n* = 3) in *Nannochloropsis oceanica* under nitrogen replete (N+) and nitrogen deplete (N−) were obtained by capillary FID-GC**.

**Lipids**	**TL**	
**Medium**	**N+**	**N−**
C14:0	5.5±0.1	5.3±0.0
C16:0	21.6±0.0	44.0±0.3
C16:1n9	5.5±0.4	2.1±0.2
C16:1n7	24.0±0.0	24.2±0.4
C16:2n4	0.5±0.0	0.3±0.0
C18:0	0.4±0.0	1.5±0.1
C18:1n9	1.8±0.0	2.9±0.1
C18:1n7	0.2±0.0	0.4±0.0
C18:2n6	2.0±0.2	1.7±0.0
C18:3n6	0.3±0.0	0.4±0.0
C18:3n3	0.6±0.1	0.0
C20:4n6	4.0±0.1	3.0±0.0
C20:5n3	30.5±0.3	14.2±0.2

*TL, total lipid*.

**Table 4:** **Fatty acid profiles of total lipids (means ± *SD, n* = 3) in *Chlorella pyrenoidosa* under nitrogen replete (N+) and nitrogen deplete (N−) were obtained by capillary FID-GC**.

**Lipids**	**TL**	
**Medium**	**N+**	**N−**
C16:0	20.4±0.0	25.6±0.0
C16:1n7	1.1±0	0.6±0
C16:3	14.2±0.0	7.6±0.0
C18:0	3.2±0	9.0±0.0
C18:1n9	5.6±0.0	21.5±0.1
C18:1n7	0.1±0	1.0±0
C18:2n6	36.7±0.0	25.5±0.1
C18:3n3	18.6±0.0	9.1±0.0

*TL, total lipid*.

This idea was further supported by the statistical analysis of the microalgal fatty acid profiles under favorable and stressed conditions across eight phyla. The candidate characteristic fatty acids were shown in bold (Supplementary Material). Due to the diversity of microalgae, both fatty acid profiles and their response to the N− were species dependent. All species accumulated SFAs or MUFAs as TAG content increased in the same manner as in *Phaeodactylum tricornutum*, except in *Porphyridium cruentum* and *Spirulina platensis*, which accumulated C18:2n6. The level of PUFAs decreased in all microalgae following the accumulation of neutral lipids, such as C18:3n3 in *Chlamydomonas* sp. JSC4, C18:4n3 in *Rhodomonas* sp., and C18:5n3 in *Gymnodinium* sp.

The accumulation of SFAs or MUFAs coupled with the decrease in the level of PUFAs was also observed under high-light or salinity stress. *Chlorella zofingiensis* accumulated a large amount of C18:1 when exposed to high-light conditions, whereas the level of the PUFA C16:3 decreased by approximately 50% (Liu et al., 2012). The level of C18:1 increased from 17.8 to 39.4% in *Sphenolithus obtusus* XJ-15 under salinity stress (Xia et al., 2013). These results revealed that a linear relationship between specific fatty acids and TAG content may also be applicable to microalgae under stress conditions.

There were some microalgae of which PUFAs such as arachidonic acid (AA, C20:4n6), EPA, and docosahexaenoic (DHA, C22:6n3) were accumulated in TAGs under stress condition. For example, the unicellular green alga *Parietochloris incise* (Trebuxiophyceae, Chlorophyta) accumulated TAGs with over 90% of total AA under nitrogen deprivation (Cohen et al., 2000; Khozin-Goldberg et al., 2002; Merzlyak et al., 2007). In the marine Haptophyte *Pavlova lutheri*, 55 and 67% of the overall cellular EPA and DHA content were attributed to the cellular TAG accumulation triggered by bicarbonate addition under nitrogen starvation (Guiheneuf and Stengel, 2013). In the red microalgae *Porphyridium cruentum* (Cohen et al., 1988) and Gracilaria (Gracilariales, Rhodophyta) (Araki et al., 1990), TAGs were predominantly constructed of AA and EPA. In this case, TAG content still could be quantified with the corresponding PUFAs, firstly to determine the quantitative correlation between them.

### Advantage of the characteristic fatty acid-based method

The approach we have proposed differed from conventional approaches. All lipid quantification data presented previously relied on “like dissolves like” with many uncertain compounds, whereas quantification of TAGs in the proposed method relied on the characteristic fatty acids, which was much easier to quantify than the entire TAG class because only one compound needs to be determined. Furthermore, the characteristic fatty acid content in total fatty acids could be determined by immediate transesterification from wet biomass. Therefore, drying the biomass and extracting the lipids could be omitted. Hence, this approach simplified sample preparation and lipid determination procedure. Furthermore, less than 5 mg of biomass is necessary due to the high sensitivity of the capillary GC-FID analysis, which is 20-times less than the amount required for conventional gravimetric determination methods. Therefore, this procedure could be used to monitor variations in lipids throughout a culture. Our procedure saved time and labor when quantifying TAGs.

In conclusion, our procedure used characteristic fatty acids to obtain lipid content and provided a unique way to quantify TAGs. The significant feature of this procedure was indirect quantification of lipids by direct detection of the individual characteristic fatty acid. Total fatty acid content of 10% and a 1.5-fold change during TAG accumulation were set as criteria to define the characteristic fatty acids. A significant linear relationship was observed between the characteristic fatty acids and TAG content. This characteristic fatty acid-based method provided a new method to quantify TAG content in different microalgal species.

## Author contributions

PS and HW designed the experiments. PS, HW, and YP performed the experiments and interpreted the data. PS and HW wrote the draft manuscript. YM established analysis methods. PW provided the subculture of the strains. SX made the critical revision of the manuscript. All the authors discussed the results and commented on the manuscript.

## Funding

This work was supported by National Oceanic Administration public welfare fund project (Grant No. 201505030); National High Technology Research and Development Program “863” (2012AA052101).

### Conflict of interest statement

The authors declare that the research was conducted in the absence of any commercial or financial relationships that could be construed as a potential conflict of interest.
