# Supplementary material for: Identification of Characteristic Fatty Acids to Quantify Triacylglycerols in Microalgae
Source: Front Plant Sci. 2016 Feb 22;7:162. doi: 10.3389/fpls.2016.00162 (PMC4761805; doi:10.3389/fpls.2016.00162)
Supplement: Supplementary file 1 [file Table1.DOCX]

Supplementary Material

Identification of characteristic fatty acids to quantify triacylglycerols in microalgae

**Pei-Li Shen^1,2†^, Hai-Tao Wang^1,2†^, Yan-Fei Pan^3^, Ying-Ying Meng^4^, Pei-Chun Wu^1^, Song Xue^1,^***

*** Correspondence:** Prof. Dr. Song Xue: email@uni.edu

# Supplementary Tables

**Supplementary Table 5.** Fatty acid profiles of different microalgae cultured under nitrogen-replete and -depleted conditions reported in literature

| **phylum** | **Species** | **Culture conditions** | **C14:0** | **C16:0** | **C16:1n7** | **C16:2n4** | **C18:0** | **C18:1n9** | **C18:1n7** | **C18:2n6** | **C18:3n3** | **C18:4n3** | **C18:5n3** | **C20:4n6** | **C20:5n3** | **C22:5n3** | **C22:6** | **Reference** |
| --- | --- | --- | --- | --- | --- | --- | --- | --- | --- | --- | --- | --- | --- | --- | --- | --- | --- | --- |
| Chlorophyta | *Chlamydomonas sp. JSC4* | N+*^1^* |  | 25.8 | 1.1 |  | 4.0 | **9.1** |  | 21.7 | **14.4** |  |  |  |  |  |  | (Ho et al., 2014) |
|  |  | N-*^2^* |  | 27.6 | 3.2 |  | 3.1 | **26.6** |  | 25.3 | **5.4** |  |  |  |  |  |  |  |
| **Eustigmatophyceae** | ***Nannochloropsis* sp.** | **N+** | **6.1** | **24.7** | **27.8** |  |  | **3.6** |  | **3.8** |  |  |  | **4.1** | **24.7** |  |  | (Dipasmita Pal and Boussiba, 2011) |
|  |  | N- | 6.4 | **40.9** | 24.8 |  |  | 6.8 |  | 2.3 |  |  |  | 3.3 | **12.1** |  |  |  |
| Haptophyta | *Isochrysis* sp. | N+ | 18.4 | 9.3 | 5.1 | 2.0 | 0.1 | **7.6** | 2.3 | 3.7 | 7.7 | **26.8** | 2.5 |  | 0.8 |  | 9.5 | (Dunstan et al., 1993) |
|  |  | N- | 16.0 | 14.1 | 2.9 | 1.1 | 0.4 | **25.6** | 1.4 | 3.7 | 4.2 | **17.1** | 1.7 |  | 0.5 |  | 6.8 |  |
| Cryptomonads | *Rhodomonas* sp. | N+ | 4.5 | **10.1** | 3.7 |  | 0.6 | 0.7 | 5.2 | 0.7 | 22 | **31.3** |  | 0.8 | 12.6 | 3.0 | 4.7 | (Huerlimann et al., 2010) |
|  |  | N- | 8.5 | **18.3** | 1.4 |  | 2.5 | 5.5 | 2.7 | 3.3 | 36.7 | **5.5** |  | 1.1 | 10.0 | 2.0 | 1.6 |  |
| Dinoflagellata | *Gymnodinium* sp. | N+ | 0.8 | 18.7 |  |  | 1.7 | 1.1 | 0.3 | 4.4 |  | 2.6 | **21.9** |  | 12.3 |  | 28.2 | (Mansour et al., 2003) |
|  |  | N- | 1.4 | 26.7 |  |  | 2.1 | 2.9 | 0.7 | 6.0 | 1.2 | 1.8 | **12.4** |  | 8.0 |  | 31.3 |  |
| Bacillariophyceae | *Phaeodactylum tricornutum* | N+ | 6.23 | 18.65 | 28.06 |  | 3.13 | 2.88 |  |  | 3.16 |  |  |  | **23.86** |  | 1.26 | (Yang et al., 2014) |
|  |  | N- | 6.36 | 26.25 | 43.18 |  | 2.4 |  |  |  | 1.27 |  |  |  | **11.84** |  | 0.65 |  |
| Rhodophyta | *Porphyridium cruentum* | N+ | 1.0 | 32.0 |  | 1.0 | 3.0 | 5.0 |  | 14.0 |  |  |  | 31.0 | 7.0 |  |  | (Breuer et al., 2012) |
|  |  | N- | 1.0 | 28.0 |  |  | 4.0 | 5.0 |  | 30.0 |  |  |  | 23.0 | 3.0 |  |  |  |
| Cyanobacteria | *Spirulina platensis* | N+ |  | 51.8 | 3.9 |  |  | 7.4 |  | 23.7 |  |  |  |  |  |  |  | (Griffiths et al., 2012) |
|  |  | N- | 2.6 | 30.8 | 6.2 |  |  | 2.1 |  | 48.1 |  |  |  |  |  |  |  |  |

*^1^*N+ = cells cultured under nitrogen-replete conditions

*^2^*N- = cells cultured under nitrogen-depleted conditions

Nitrogen-replete and nitrogen-depleted conditions were specific to the species as described in the cited articles, and represented normal and stressed conditions, respectively.

# References

Breuer, G., Lamers, P.P., Martens, D.E., Draaisma, R.B., and Wijffels, R.H. (2012). The impact of nitrogen starvation on the dynamics of triacylglycerol accumulation in nine microalgae strains. *Bioresour Technol* 124**,** 217-226.

Dipasmita Pal , I.K.-G., Zvi Cohen ,, and Boussiba, S. (2011). The effect of light, salinity, and nitrogen availability on lipid production by *Nannochloropsis sp*. *Appl Microbiol Biotechnol***,** 1429–1441.

Dunstan, G., Volkman, J., Barrett, S., and Garland, C. (1993). Changes in the lipid composition and maximisation of the polyunsaturated fatty acid content of three microalgae grown in mass culture. *Journal of Applied Phycology* 5**,** 71-83.

Griffiths, M., Van Hille, R., and Harrison, S. (2012). Lipid productivity, settling potential and fatty acid profile of 11 microalgal species grown under nitrogen replete and limited conditions. *Journal of Applied Phycology* 24**,** 989-1001.

Ho, S.-H., Nakanishi, A., Ye, X., Chang, J.-S., Hara, K., Hasunuma, T., and Kondo, A. (2014). Optimizing biodiesel production in marine *Chlamydomonas sp JSC4* through metabolic profiling and an innovative salinity-gradient strategy. *Biotechnology for Biofuels* 7.

Huerlimann, R., De Nys, R., and Heimann, K. (2010). Growth, lipid content, productivity, and fatty acid composition of tropical microalgae for scale-up production. *Biotechnol Bioeng* 107**,** 245-257.

Mansour, M.P., Volkman, J.K., and Blackburn, S.I. (2003). The effect of growth phase on the lipid class, fatty acid and sterol composition in the marine dinoflagellate, *Gymnodinium sp.* in batch culture. *Phytochemistry* 63**,** 145-153.

Yang, Z.-K., Ma, Y.-H., Zheng, J.-W., Yang, W.-D., Liu, J.-S., and Li, H.-Y. (2014). Proteomics to reveal metabolic network shifts towards lipid accumulation following nitrogen deprivation in the diatom *Phaeodactylum tricornutum.* *Journal of Applied Phycology* 26**,** 73-82.
